# The Diagnostic Role of FGF 21 in Endometrial Cancer and Other Pathologies of the Uterine Corpus

**DOI:** 10.3390/diagnostics13030399

**Published:** 2023-01-22

**Authors:** Anna Jagodzińska, Anita Chudecka-Głaz, Kaja Michalczyk, Ewa Pius-Sadowska, Sylwia Wieder-Huszla, Anna Jurczak, Bogusław Machaliński

**Affiliations:** 1 Department of Gynecological Surgery and Gynecological Oncology of Adults and Adolescents, Pomeranian Medical University, 70-204 Szczecin, Poland; 2 Department of General Pathology, Pomeranian Medical University in Szczecin, al. Powstańców Wielkopolskich 72, 70-111 Szczecin, Poland; 3 Department of Clinical Nursing, Pomeranian Medical University in Szczecin, 71-210 Szczecin, Poland

**Keywords:** endometrial cancer, FGF 21, CA 125, HE4, obesity

## Abstract

Endometrial cancer is becoming an increasing problem. Taking into account its pathomechanisms, we aimed to investigate whether FGF 21, an important metabolism regulator, could be used as a biomarker for endometrial cancer. The study included 233 patients who were classified into five subgroups depending on the result of the histological examination: endometrial carcinomas, sarcomas, endometrial polyps, fibroids, and normal endometrium. Statistically significantly higher FGF 21 levels were found in patients diagnosed with malignant lesions (*p* < 0.001). FGF 21 concentration correlated with the degree of cellular differentiation (*p* = 0.020) and the presence of lymph node metastases (*p* = 0.009). The diagnostic performance characteristics of FGF 21 as an EC diagnostic marker demonstrated an AUC of 0.677. Of all of the assessed biomarkers, FGF 21 had the highest specificity (90%), yet limited sensitivity (41%). Additionally, HE4 and CA 125 were confirmed to have roles as EC biomarkers, with a higher accuracy for HE4 (79% vs. 72%).

## 1. Introduction

Endometrial cancer (EC) is one of the most common gynecological malignancies in the world. In 2020, 417,367 [1] patients were newly diagnosed, mostly affecting postmenopausal women. EC is only hereditary in approximately 10% of cases, while the majority are associated with hormonal imbalance [2]. Taking into account an increasing life expectancy and the greater prevalence of obesity, hypertension, diabetes, and insulin resistance, which are the main risk factors for endometrial cancer, it can be assumed that the number of EC diagnoses will continue to rise. Despite the proven associations between metabolic syndrome and endometrial cancer pathogenesis, the exact molecular mechanism remains unknown. 

FGF 21 (fibroblast growth factor 21) is a protein formed by 210 amino acids that belongs to intracellular FGFs. It is synthesized by hepatocytes, white adipose tissue, pancreatic, and skeletal muscle cells, and plays a role in the body’s energy metabolism, demonstrating pleiotropic effects [3,4]. Unlike most of the FGFs, which are sequestered to the extracellular matrix, FGF 21 has reduced affinity for heparan sulfate glycosaminoglycans, and diffuses into circulation acting on distal tissues [5,6,7]. The main mechanism of FGF 21 action is based on its function to regulate glucose and lipid metabolism. Elevated circulating levels of FGF 21 were demonstrated in multiple pathophysiological conditions, including obesity [8,9,10,11], type 2 diabetes [12,13], hepatosteatosis [9,14,15], and pancreatitis [16,17].

It has been demonstrated that in the states of impaired glucose tolerance and type 2 diabetes, the concentration of FGF 21 increases; however, the concentration was found to be lower in patients with type 1 diabetes. Its level correlates with the level of glucose and glycosylated hemoglobin during fasting periods and is inversely proportional to the body’s sensitivity to insulin [7]. Higher FGF 21 levels were also noticed in obese patients, probably due to its production by adipose tissue. During periods of fasting, when energy metabolism is converted into the oxidation of free fatty acids, the hepatic synthesis of FGF 21 increases [18]. Its action prevents dyslipidemia and lipid accumulation in pancreatic cells and hepatocytes, and thus fatty pancreas and liver [19,20]. 

Taking into account the spectrum of action of FGF 21 in glucose and lipid metabolism, and the risk factors for endometrial cancer, we aimed to assess this protein’s diagnostic and prognostic value.

## 2. Materials and Methods

The study included 223 patients treated at the Department of Gynecological Surgery and Gynecological Oncology of Adults and Adolescents. Patients were admitted for surgical treatment due to abnormal vaginal bleeding, abnormal histological results, and/or abnormal ultrasound imaging. The study was approved by the Bioethics Committee of the Pomeranian Medical University in Szczecin. Informed consent to participate in the study was obtained from all patients. For the purpose of the study, a 5 mL blood sample was taken from each patient at the time of admission for surgical treatment. The samples were centrifuged and properly stored at −20 °C until further analysis. Each of the study participants was anonymously described in terms of age, height, weight, BMI, and comorbidities such as hypertension, coronary artery disease, diabetes, hypothyroidism, CA 125 and HE4 levels. Serum CA 125 and HE4 levels were assessed as a part of routine examination at the time of patient admission. Moreover, for patients diagnosed with malignant lesions, FIGO staging and the degree of cellular differentiation were determined. Based on the results of the histological examination, the patients were divided into five groups: endometrial carcinomas, endometrial polyps, normal endometrium, myomas, and sarcomas.

Plasma FGF 21 concentration was determined using multiplex fluorescent-bead-based immunoassays (LUMINEX Corporation, Austin, TX, USA) and the xPONENT 3.1 software on MILLIPLEX panels in accordance with the manufacturers’ protocol. 

Statistical analysis was performed to compare the obtained results between the assessed groups in terms of the following variables: age, weight, BMI, CA 125, HE4, and FGF 21 levels. The incidence of comorbidities was assessed. Patients were assigned to subgroups A, B, and C depending on their BMI:

1. A—BMI < 29.9—underweight, normal weight, and overweight
2. B—BMI 30–34.9—obese class I
3. C—BMI > 35—obese class II and III

Subgroups A, B, and C were compared in terms of the described variables and disease frequency.

The group of patients diagnosed with endometrial cancer was characterized in terms of the examined variables and tumor characteristics, including the degree of cellular differentiation, histological type, lymph node involvement, blood vessel infiltration, and recurrence of the disease. For the basic descriptive analysis of the examined groups, data were reported as mean ± standard deviation or median (first quartile; third quartile). The groups were compared using the Mann–Whitney *U* test, while for independent groups Student’s *t*-test was used. In cases where more than two groups were compared, Kruskal–Wallis and Anova-Welch tests were used. The association of categorical variables was assessed using the Pearson Chi-squared test and Fisher test. The receiver operating characteristic (ROC) was used to assess the sensitivity and specificity of tested biomarkers. Statistical analysis was performed with the assumption of alpha = 0.05. All calculations were performed using R Statistical Analysis Software, R Foundation for Statistical Computing, Vienna, Austria (version R-4.1.2.).

## 3. Results

### 3.1. Group Characteristics

The analysis was based on 223 patients. Patients with endometrial cancer lesions constituted the largest group (44.8%), while women diagnosed with polyps or normal endometrial tissue were half as numerous (22.0% and 21.1%, respectively). The smallest groups of patients were those diagnosed with fibroids (8.5%) and sarcomas (3.6%, 8 patients). Group characteristics are presented in Table 1. 

The highest mean age was observed for patients diagnosed with endometrial cancer (65.59 ± 12.40 years), while women in the sarcoma group were on average 60.75 ± 10.98 years old. Patients diagnosed with normal endometrial tissue and endometrial polyps were of a relatively similar age (56.06 ± 8.71 years and 55.06 ± 13.43 years, respectively). The youngest mean age was observed for the myoma group (44.58 ± 7.54 years).

Among the analyzed variables, significant differences between the groups were found for patients’ age and biomarker concentration. The highest FGF 21 levels were noticed among sarcoma patients (median = 77.59 pg /mL), while the median FGF 21 value in EC patients was 62.28 pg /mL. About two-fold lower median values were obtained among the three remaining groups of patients diagnosed with benign lesions (37.36 pg/mL for polyps, 32.93 pg/mL for fibroids, and 31.94 pg/mL for normal endometrium).

As a part of the study, we also assessed the commonly used markers for endometrial cancer, i.e., CA 125 and HE4. The highest CA 125 levels were found among women diagnosed with sarcomas (median = 43.15 U/mL). The marker was two-fold lower in endometrial cancer patients (median = 26.55 U/mL), and the lowest values were noticed for patients with a histological finding of normal endometrial tissue (median = 12.20 U/mL). 

Statistically significant differences between the assessed groups were also found in HE4 concentration. HE4 levels were the highest in the sarcoma group (median = 90.30 pmol/L). The median value of HE4 in EC patients was determined to be 77.35 pmol/L. In patients with normal endometrium and polyps, HE4 levels were similar and equaled 51.75 pmol/l and 49.90 pmol/L, respectively (see Table 2).

We also tried to assess if there were any differences in patients’ weight and BMI distribution; however, we found no significant differences in group distribution (*p* = 0.869 and *p* = 0.157, respectively) (Table 2).

For the purpose of the study, we also assessed for the presence of any comorbidities. The most common was hypertension, noted in almost 43.5% of the studied population. Patients also frequently reported thyroid diseases (14.3%) and diabetes (11.7%). Coronary heart disease was reported by four patients. The distribution of comorbidities is presented in Table 3. Statistically significant differences in terms of diabetes incidence were found between the analyzed groups (*p* = 0.025). Its incidence was the highest in the sarcoma group (25.0), while in the women with normal endometritis and polyps, diabetes occurred in individual cases (three patients). 

### 3.2. Group Characteristics of Patients Diagnosed with Malignant Neoplasms

Among the histological types of endometrial cancer, endometrioid EC was the most frequently diagnosed (79.6%). The serous type was found in 9.3% of patients, while the remaining histological types occurred in isolated cases. 

The majority of tumor tissues were found to be grade 2 (49.1%), followed by grade 1 (25.9%). In terms of FIGO classification, most of the patients were diagnosed with FIGO IA (41.7%), followed by IB (27.8%) and II (11.1%), accounting for 79.7% of all patients with endometrial malignancies. Vascular infiltration was detected in eight patients (7.4%). Metastases were found in seven patients (6.5%) (Table 4).

### 3.3. Correlations between FGF 21, CA 125, and HE4 and Patients’ Body Weight and BMI

For the purpose of statistical analysis, we subdivided the patients based on their weight status into three subgroups: 

A—BMI < 29.9—underweight, normal weight, and overweight

B—BMI 30—34.9—obese class I

C—BMI > 35—obese class II and III

The concentrations of FGF 21, CA 125, and HE4 were analyzed in terms of body weight categories. Statistical analyses were performed for each of the histological findings separately, excluding sarcomas (due to limited population sample). The results are presented in Table 5. 

We found no correlation between the levels of selected proteins and endometrial cancer when differentiated into specific body weight groups. A significant impact of body weight on FGF 21 concentration was found in patients with endometrial polyps, as patients with greater body mass tended to have higher FGF 21 concentration. 

### 3.4. Assessment of the Relationship between the Levels of FGF 21, CA 125, and HE4 and Unfavorable EC Prognostic Factors

In this part of the study, we only analyzed patients diagnosed with endometrial cancer. Patients’ age, weight, BMI, and concentrations of the studied proteins were compared in terms of cellular differentiation (grading), histological subtypes, presence of metastases in the iliac lymph nodes, EC recurrence, and FIGO classification (Table 6). 

We found no statistically significant difference in mean age, weight, and BMI level for any of the analyzed subgroups (*p* > 0.05). There was also no significant relationship between the specific subgroups and CA 125 concentration (*p* > 0.05). However, we found FGF 21 concentration to be influenced by tumor grade (*p* = 0.020 for grade 1 vs. 3), and the presence of metastases within the iliac lymph nodes (*p* = 0.009). Significantly higher levels of FGF 21 were found in patients with grade 3 EC when compared to patients with grade 1, MD = −57.91, CI95 [−105.42; −8.31]. Higher FGF 21 levels were also observed among patients with iliac lymph node metastases, MD = 128.83, CI95 [21.64; 377.52]. Moreover, for HE4, a significant correlation was demonstrated for tumor grading (p = 0.011). Patients with higher tumor grading were found to present with higher HE4 levels, MD = −120.20, CI95 [−196.70; −16.90] (see Table 6).

### 3.5. The Assessment of FGF 21, CA 125, and HE4 as Diagnostic Biomarkers 

As a part of the study, we assessed FGF 21, CA 125, and HE4 diagnostic potential. For the purpose of the study, we used patients with normal endometrial tissue, endometrial polyps, and uterine fibroma as a control group. HE4 was found to have the highest diagnostic potential (AUC = 0.828). The optimal cut-off point was determined at 58.05 pmol/L. The AUC for CA 125 was found to equal 0.751, while FGF 21 was found to have a moderate diagnostic potential (AUC = 0.677). 

**Figure 1:**
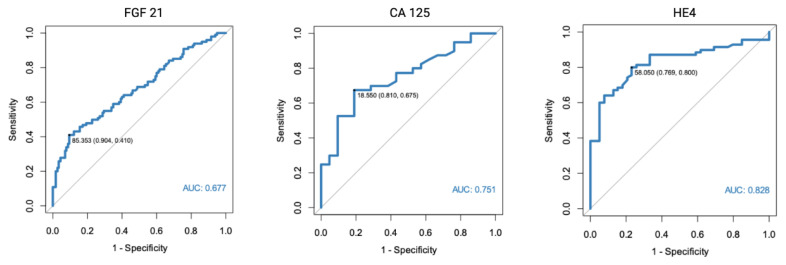
ROC curve analysis of FGF 21, CA 125, and HE4 performance in EC diagnostics (endometrial cancer vs. normal endometrium, polyps, and fibromas).

**Figure 2:**
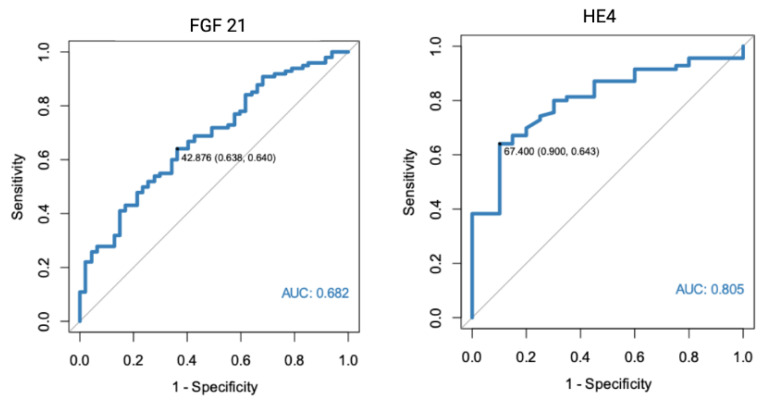
ROC curve analysis of FGF 21 and HE4 performance in EC diagnostics (endometrial cancer vs. normal endometrium).

**Figure 3:**
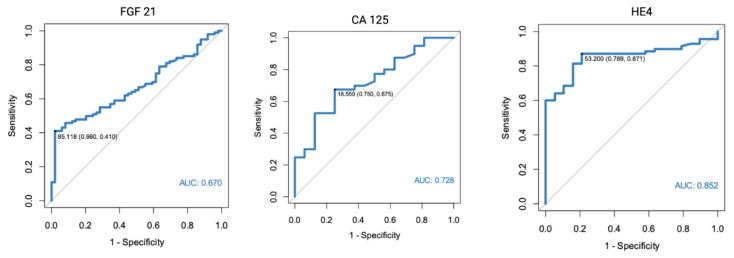
ROC curve analysis of FGF 21, CA 125, and HE4 performance in EC diagnostics (endometrial cancer vs. endometrial polyps).

In a separate analysis, where the control group consisted only of patients with histologically confirmed normal endometrial tissue, significant results were found for both FGF 21 (*p* < 0.001, AUC = 0.682) and HE4 (*p* < 0.001, AUC = 0.805). All of the markers were also found to have diagnostic potential in the differentiation between endometrial cancer and benign conditions, using endometrial polyps as a control group. HE4 had the highest diagnostic value with an AUC equal to 0.852. Receiver-operating characteristics curve analysis of the specific markers are presented in Figure 1, Figure 2 and Figure 3.

### 3.6. The Assessment of Diagnostic Performance Characteristics of FGF 21, CA 125, and HE4 Accounting for Tumor Characteristics

As a part of the study, we conducted multiple analyses accounting for different tumor characteristics, including tumor grading, histology, and FIGO staging. The diagnostic potential of FGF 21, CA 125, and HE4 was assessed using ROC analysis.

None of the tests were able to differentiate between tumor grading (*p* > 0.05) (Table 7). The diagnostic performance characteristic of FGF 21 for histology differentiation between endometrioid and non-endometrioid histology demonstrated a sensitivity of 21% and specificity of 100% for endometrioid tumor diagnosis. The AUC was 0.597.

The diagnostic accuracy of selected tumor markers was also analyzed by FIGO stage, demonstrating the potential of FGF 21 to differentiate between different FIGO stages in early endometrial cancer (FIGO IA + IB) and EC higher staging (AUC 0.602, *p* = 0.004). However, the marker had limited sensitivity (0.53) and specificity (0.65).

Moderate FGF 21 diagnostic potential (AUC 0.602, *p* = 0.004) was found for differentiation between FIGO staging. A 44% PPV and 73% NPV, with a sensitivity of 53% and specificity of 65%, was found to detect early endometrial cancer (FIGO IA and IB).

## 4. Discussion

Abnormal uterine bleeding is one of the most common causes of gynecological referrals among postmenopausal patients. However, it is also one of the first symptoms of uterine cancer, which often allows early patient diagnosis. 

Endometrial cancer diagnosis and treatment qualification (surgical and/or adjuvant treatment) are made based on the histology results, obtained from endometrial biopsy or curettage of the uterine cavity. However, the obtained exam results, even with the addition of diagnostic imaging, do not always allow for the unequivocal selection of patients with poor prognosis. The new endometrial cancer molecular classification has identified four categories of endometrial carcinomas, differentiating tumors based on their clinical, pathologic, and molecular features (POLE ultra-mutated, microsatellite instability (MSI)/hypermutated, copy number low/microsatellite stable, and serous-like/copy number high [21]). Each subgroup, based on its characteristic features, allows better patient selection and stratification; yet, in everyday practice, the method is still limited due to its cost and complexity. Even though molecular classification has made significant refinements to the diagnostics and prognostics of endometrial cancer, efforts to identify new diagnostic and prognostic markers or their combinations should be continued. 

In our research, we investigated whether FGF 21 can be used as a diagnostic marker for endometrial cancer. For the purpose of the study, we divided the patients into specific groups based on their histology findings (endometrial cancer, sarcoma, normal endometrium, endometrial polyp, and myoma). Benign lesions and normal endometrium were treated as a control group. To investigate the whole spectrum of endometrial pathologies, sarcomas were also included in the study; however, due to rare findings, the group consisted of only eight patients. The study groups did not differ significantly in terms of body weight, BMI, or the prevalence of chronic diseases (thyroid disease, hypertension, coronary artery disease), except for diabetes, which was the most frequently diagnosed among sarcoma and endometrial cancer patients. A statistically significant difference between the groups was also noted for patients’ age, with the oldest patients being among the EC group.

In order to evaluate FGF 21 as an endometrial cancer marker, we compared its diagnostic value with CA 125 and HE4, previously described markers, whose role in the diagnostic and prognostic process is well documented [22,23,24,25,26,27,28]. Our statistical analysis showed that FGF 21 is statistically significantly correlated with endometrial malignant neoplasms. The median value for FGF 21 equaled 77.59 pg/mL in the sarcoma group and 62.58 pg/mL for endometrial cancer. The values were almost twice as high as in the control group, with median for polyps—37.36 pg/mL, fibroids—32.93 pg/mL, and normal endometrium—31.94 pg/mL. The findings were similar for CA 125 and HE4, as their concentrations were also the highest among patients diagnosed with sarcomas and carcinomas (CA 125, 43.15 U/mL and 26.55, respectively; HE4, 90.3 pmol/L, and 77.35 pmol/L, respectively). 

The use of FGF 21 in EC diagnosis was also investigated by Cymbaluk-Płoska et al. [29]. Even though both studies were performed at the same center, they concerned different groups of patients and were performed independently on different patient groups. The authors conducted their analysis on a group of 182 patients who underwent surgical treatment for abnormal vaginal bleeding, of whom 98 were diagnosed with endometrial cancer. The remaining patients were found to have endometrial polyps or normal endometrium histology (33 and 51 patients, respectively). The authors obtained similar results, as median FGF 21 for EC patients was 181.8 pg/mL, and 152.1 pg/mL in the control group (normal endometrium and polyps marked together).

Endometrial cancer is frequently associated with obesity and metabolic syndrome. Previous studies have demonstrated a 2.45-fold increase of EC risk in overweight/obese patients and a 2.12-fold risk increase among patients suffering from diabetes [30]. The characteristics of metabolic syndrome that may favor cancer formation and create a pro-oncogenic status include increased proinflammatory cytokine status [20,31], adipokine regulation imbalance [32,33], hormonal dysregulation including hyperestrogenism [34], disturbances to tissue microenvironment, insulin resistance, and hyperinsulinemia [35]. 

FGF 21 is a hepatokine that takes part in glucose and lipid metabolism. Elevated circulating levels of FGF 21 were demonstrated in multiple pathophysiological conditions, including obesity [8,9,10], type 2 diabetes [12,13], hepatosteatosis [9,14,15,36], and pancreatitis [16,17]. However, considering the effect of FGF 21 that causes reduction of fasting plasma glucose, triglyceride and insulin levels [37,38,39,40], the increased FGF 21 concentration in obese patients seems paradoxical. Previous researchers have tried to explain this paradigm as a FGF 21-resistant state or due to the circulatory form of FGF 21 being nonfunctional due to its proteolytic processing [5]. Further studies examining the role of FGF 21 in obesity and obesity-related conditions are needed. 

In the presented study, we found no statistically significant relationship between FGF 21, CA 125, and HE4 concentration, and patient body weight or BMI status. Despite FGF 21 concentration being higher among overweight and obese patients, the correlation was insignificant (*p* = 0.392). The tendency of our results is similar to that obtained by Cymbaluk-Płoska et al. [29], who found a significant correlation between FGF 21 concentration and obesity prevalence (*p* = 0.001). 

As a part of the study, we also tried to determine the influence of EC prognostic characteristics, such as tumor grading, histological type, presence of lymph node metastases, vascular infiltration, recurrence, and FIGO staging on FGF 21. We found that FGF 21 concentration correlated with tumor grade (1 vs. 3) (*p* = 0.02) and metastases to the iliac lymph nodes (*p* = 0.009). Additionally, HE4 level correlated with tumor grading, and higher HE4 levels were observed among patients with grade 3 tumors. 

Finally, we assessed the endometrial cancer diagnostic potential of FGF 21, CA 125, and HE4 using ROC analysis. Using all of the assessed benign uterine lesions as a control group (normal endometrium, polyps, and fibromas), the optimal cut-off point was established at 85.35 pg/mL. The diagnostic performance characteristics of FGF 21 as an EC diagnostic marker demonstrated a PPV of 0.79 and NPV of 0.64. Of all of the assessed biomarkers, FGF 21 had the highest specificity (90%), yet very limited sensitivity (41%). The AUC was 0.677. Similar results were found when only patients with normal endometrial histology or endometrial polyps were used separately as a control group. The specificity of the FGF 21 marker was even higher when only endometrial polyps were used as the control group (98%). 

The diagnostic performance characteristics were also calculated for CA 125 and HE4. The highest AUC was found for HE4 in a setting where only endometrial polyps were used as the control group (AUC 0.852, 95%Cl 0.772–0.932), with 87% sensitivity and 79% specificity. However, it should be noted that a slightly different cut-off point was used than when assessed for all benign endometrial lesions in the control group. 

We also assessed the diagnostic performance characteristics for the use of FGF 21 in patient stratification based on endometrial cancer prognostic factors, including tumor differentiation, histology, and FIGO staging. Vascular infiltration and presence of lymph node metastases were excluded from the analysis due to an insufficient number of data. FGF 21 was found to be able to differentiate between endometrioid and non-endometrioid histology of EC (AUC 0.597 for serous tumors as the control group and AUC 0.568 for tumor histology other than endometrioid). None of the tested markers proved useful in tumor-grading determination. 

The diagnostic accuracy of selected tumor markers was also analyzed by FIGO stage, demonstrating the potential of FGF 21 to differentiate between FIGO early endometrial cancer (FIGO IA + IB) and higher EC staging (AUC 0.602, *p* = 0.004). However, the marker had limited sensitivity (0.53) and specificity (0.65). Moreover, it should be noted that the majority of patients included in the analysis were diagnosed at an early stage of the disease and only 25 patients were found to have FIGO > IB, thus limiting the sample population. 

When assessing test performance, it is necessary to evaluate test characteristics using a cut-off point, above which the test is abnormal and below which it is assessed as normal. We decided not to use standard cut-off points for the commonly used markers (≥35 U/mL for CA 125 and ≥70 pmol/L for HE4) but to use the calculated values. However, as different cut-off values were established for tests using different populations as control groups, it is misleading to compare the PPV and NPV between the groups. Additionally, the higher incidence of malignant tumors compared to the general population might have affected test sensitivity and specificity, resulting in higher sensitivity and lower specificity than expected. 

As a part of the study, we analyzed the diagnostic potential of CA 125, HE4, and FGF 21 separately, and did not check for any combinations between the selected markers. Future studies should be conducted to assess if any combination results in better diagnostic potential.

## 5. Conclusions

In conclusion, the presented results provide further evidence for the use of CA125, HE4, and FGF 21 as endometrial cancer biomarkers. In our study, we did not confirm the relationship between patients’ body weight and FGF 21 concentration. 

## Figures and Tables

**Table 1:** Group characteristics.

Variable	n (%)
Total population	223 (100)
Endometrial cancer	100 (44.8)
Endometrial polyp	49 (22.0)
Normal endometrial tissue	47 (21.1)
Myoma	19 (8.5)
Sarcoma	8 (3.6)

n (%)—number of patients (% of the assessed population).

**Table 2:** Comparison of the selected variables between the study groups.

Variable	Endometrial Cancer	Sarcoma	Normal Endometrium	Endometrial Polyp	Myoma	*p*
Age [years] *	65.59 ± 12.40	60.75 ± 10.98	56.06 ± 8.71	55.06 ± 13.43	44.58 ± 7.54	<0.001 ^2^
Weight [kg]	76.44 ± 15.38	73.38 ± 14.20	77.73 ± 15.45	74.74 ± 13.46	73.41 ± 16.35	0.869 ^1^
BMI [kg/m2]	29.59 ± 6.04	28.71 ± 6.17	30.98 ± 7.20	27.81 ± 5.79	26.59 ± 5.63	0.157 ^1^
FGF 21 [pg/mL]	62.28 (28.92; 113.16)	77.59 (46.56; 112.49)	31.94 (13.14; 59.15)	37.36 (21.67; 59.65)	32.93 (13.51; 66.71)	<0.001
CA 125 [U/mL]	26.55 (16.23; 81.08)	43,15 (21,.8;56.10)	12.20 (12.10; 15.70)	15.55 (11.22; 19.47)	-	0.008
HE4 [pmol/L]	77.35 (61.98; 152.23)	90.30 (61.85; 173.12)	51.75 (43.67; 62.15)	49.90 (47.15; 52.70)	-	<0.001

Data are reported as mean ± standard deviation or median (first quartile; third quartile). The groups were compared using Kruskal–Wallis, Anova ^1^ and Anova Welch ^2^. * at the time of diagnosis.

**Table 3:** Distribution of comorbidities between the analyzed groups.

Variable	Endometrial Cancer (n = 100)	Sarcoma (n = 8)	Normal Endometrium (n = 47)	Polyp (n = 49)	Fibroma (n = 19)	*p*
Thyroid disease	15 (15.0)	0 (0.0)	7 (14.9)	6 (12.2)	4 (21.1)	0.731 ^1^
Hypertension	58 (58.0)	5 (62.5)	17 (36.2)	15 (30.6)	2 (10.5)	0.680
Diabetes	18 (18.0)	2 (25.0)	3 (6.4)	3 (6.1)	0 (0.0)	0.025
Coronary disease	6 (6.0)	0 (0.0)	2 (4.3)	1 (2.0)	0 (0.0)	0.106

n—number of patients (%). Pearson Chi-squared test and Fisher test were used ^1.^

**Table 4:** Characteristics of endometrial cancer and sarcoma patients in terms of selected prognostic factors.

Variable	n (%)
Total number of patients	108 (100)
Tumor histology	
Endometrioid	86 (79.6)
Serous	10 (9.3)
Other	12 (11.1)
Grade	
1	28 (25.9)
2	53 (49.1)
3	17 (15.7)
FIGO classification	
I	1 (0.9)
IA	45 (41.7)
IB	30 (27.8)
II	12 (11.1)
IIB	1 (0.9)
III	1 (0.9)
IIIA	4 (3.7)
IIIC	1 (0.9)
IIIC1	1 (0.9)
IIIC2	1 (0.9)
IV	3 (2.8)
Vascular infiltration	
Yes	8 (7.4)
No	32 (29.6)
Metastasis	
Yes	7 (6.5)
No	48 (44.4)

n (%)—number of patients (% of patients).

**Table 5:** Comparison of variable distributions between different tumor histologies, accounting for patients’ body weight.

Variable	BMI	Endometrial Cancer	Normal Endometrium	Polyp	Fibroma
FGF 21 [pg/mL]	A	50.54 (25.60; 98.99)	44.31 (30.43; 89.79)	34.21 (21.56; 47.90)	31.82 (11.66; 50.68)
	B	80.77 (24.59; 126.86)	34.55 (34.55; 34.55)	83.56 (60.23; 84.26)	87.16 (24.60; 91.59)
	C	85.84 (49.38; 101.64)	101.48 (73.41; 117.91)	53.62 (44.93; 64.90)	49.00 (49.00; 49.00)
*p*		0.392	0.119	0.003	0.319
CA 125 [U/mL]	A	32.30 (16.95; 165.70)	16.50 (16.10; 16.90)	16.40 (11.80; 20.75)	-
	B	20.10 (16.10; 50.58)	-	16.05 (13.55; 34.05)	-
	C	19.25 (11.95; 33.92)	11.35 (10.93; 11.77)	8.20 (8.20; 8.20)	-
*p*		0.523	0.333 ^1^	0.468	-
HE4 [pmol/L]	A	78.40 (61.70; 174.00)	56.05 (56.02; 56.08)	48.60 (44.02; 50.77)	-
	B	76.20 (59.80; 115.70)	41.50 (41.50; 41.50)	52.70 (51.25; 57.08)	-
	C	74.95 (65.30; 86.17)	65.70 (55.05; 76.35)	66.80 (66.80; 66.80)	-
*p*		0.863	0.368	0.069	-

A—underweight, normal weight, and overweight; B—obese class I; C—obese class II and III. Data were noted as mean ± standard deviation or median (first quartile; third quartile). Group comparison was performed with the use of Mann–Whitney U tests ^1^.

**Table 6:** Comparison of the selected variables among endometrial cancer patients.

	Age [years] *	Weight [kg]	BMI [kg/m^2^]	FGF 21 [pg/mL]	CA 125 [U/mL]	HE4 [pmol/L]
Grade						
1	62.86 ± 13.65	76.56 ± 16.25	29.47 ± 6.54	56.63 (28.85; 108.64)	19.40 (16.10; 52.77)	67.50 (59.80; 93.80)
2 + 3	66.24 ± 11.86	76.57 ± 15.64	29.64 ± 6.08	65.14 (31.65; 110.25)	30.60 (15.88; 95.38)	81.20 (62.60; 159.25)
MD (95% CI)	−3.38 (−8.93; 2.16) ^1^	−0.01 (−7.30; 7.28) ^1^	−0.18 (−3.04; 2.69) ^1^	−8.51 (−26.85; 20.12)	−11.20 (−33.50; 6.30)	−13.70 (−35.90; 5.80)
*p*	0.229 ^2^	0.998 ^2^	0.904 ^2^	0.848	0.501	0.167
Grade						
1	62.86 ± 13.65	76.56 ± 16.25	29.47 ± 6.54	56.63 (28.85; 108.64)	19.40 (16.10; 52.77)	67.50 (59.80; 93.80)
3	70.25 ± 12.46	78.07 ± 13.51	30.06 ± 5.79	114.54 (69.51; 196.77)	55.20 (25.45; 204.95)	187.70 (112.90; 261.70)
MD (95% CI)	−7.39 (−15.77; 0.98) ^1^	−1.51 (−11.56; 8.54) ^1^	−0.60 (−4.72; 3.52) ^1^	−57.91 (−105.42; −8.31)	−35.80 (−197.40; 4.30)	−120.20 (−196.70; −16.90)
*p*	0.082 ^2^	0.763 ^2^	0.772 ^2^	**0.020**	0.149	**0.011**
Tumor histology						
Endometrioid	64.92 ± 11.98	76.23 ± 15.01	29.59 ± 6.01	58.57 (28,64; 107.29)	23.55 (15.65; 73.65)	76.25 (62.52; 144.07)
Serous	72.20 ± 14.75	73.11 ± 15.24	28.47 ± 5.88	78,45 (44.66; 129.15)	77.70 (46.13; 148.15)	111.40 (53.30; 174.00)
MD (95% CI)	−7.28 (−15.42; 0.86) ^1^	3.12 (−7.36; 13.59) ^1^	1.12 (−3.06; 5.30) ^1^	−19.88 (−66.72; 19.03)	−54.15 (−134.60; 54.40)	−35.15 (−92.90; 65.20)
*p*	0.079 ^2^	0.556 ^2^	0.596 ^2^	0.322	0.243	0.917
Tumor histology						
Endometrioides	64.92 ± 11.98	76.23 ± 15.01	29.59 ± 6.01	58.57 (28.64; 107.29)	23.55 (15.65; 73.65)	76.25 (62.52; 144.07)
Other	69.71 ± 14.56	77.81 ± 18.24	29.59 ± 6.51	78.45 (33.31; 129.15)	77.70 (46.13; 148.15)	96.30 (60.27; 158.35)
MD (95% CI)	−4.80 (−11.86; 2.27) ^1^	−1.58 (−10.73; 7.57) ^1^	−0.01 (−3.60; 3.59) ^1^	−19.88 (−57.34; 18.71)	−54.15 (−134.60; 54.40)	−20.05 (−61.10; 58.80)
*p*	0.181 ^2^	0.733 ^2^	0.997 ^2^	0.418	0.243	0.975
metastasis to illiac lymphnodes						
Yes	70.86 ± 9,55	74.83 ± 9.04	29.00 ± 3.43	182.36 (96.19; 421.00)	43.95 (29.27; 58.62)	76.30 (74.55; 116.30)
No	64.36 ± 11,99	75.19 ± 14.87	29.07 ± 5.39	53.53 (24.51; 95.48)	19.00 (11.00; 33.45)	65.30 (56.30; 98.85)
MD (95% CI)	6.50 (−3.06; 16.07) ^1^	−0.36 (−12.92; 12.21) ^1^	−0.07 (−4.63; 4.50) ^1^	128.83 (21.64; 377.52)	24.95 (−142.80; 63.80)	11.00 (−67.20; 92.80)
*p*	0.178 ^2^	0.955 ^2^	0.977 ^2^	**0.009**	0.513	0.369
Angioinvasion						
Yes	72.86 ± 12,77	70.86 ± 10.49	28.06 ± 4.46	85.84 (39.71; 421.00)	32.30 (32.30; 32.30)	76.00 (76.00; 76.00)
No	63.97 ± 10,89	78.80 ± 17.77	30.46 ± 6.61	77.35 (24.95; 115.24)	15.30 (12.40; 18.80)	65.30 (60.02; 77.00)
MD (95% CI)	8.89 (−0.69; 18.47) ^1^	−7.94 (−22.21; 6.33) ^1^	−2.40 (−7.76; 2.96) ^1^	8.49 (−37.47; 377.52)	17.00 (−22.90; 22.80)	10.70 (−258.70; 56.60)
*p*	0.068 ^2^	0.266 ^2^	0.370 ^2^	0.350	0.545	0.562
Recurrence						
Yes	70.33 ± 9.20	65.67 ± 11.33	25.69 ± 4.82	41.34 (21.34; 61.53)	174.00 (174.00; 174.00)	166.00 (83.30; 174.50)
No	65.42 ± 11.69	67.00 ± 10.84	26.16 ± 3.51	37.10 (25.69; 80.73)	23.10 (18.25; 63.58)	82.15 (73.60; 165.50)
MD (95% CI)	4.92 (−6.71; 16.55) ^1^	−1.33 (−12.98; 10.32) ^1^	−0.48 (−4.68; 3.73) ^1^	4.24 (−57.39; 28.98)	150.90 (−118.00; 164.50)	83.85 (−116.40; 105.60)
*p*	0.383 ^2^	0.811 ^2^	0.813 ^2^	0.553	0.444	0.594
FIGO						
IA	63.67 ± 12,34	77.94 ± 15.18	30.05 ± 5.84	59.74 (21.74; 113.18)	17.60 (12.60; 30.60)	65.30 (54.60; 86.50)
IB	69.50 ± 10,84	72.75 ± 15.60	28.44 ± 6.08	54.89 (37.01; 79.04)	32.30 (26.50; 49.00)	82.80 (68.50; 162.20)
MD (95% CI)	−5.83 (−11.49; −0.17) ^1^	5.19 (−2.29; 12.66) ^1^	1.60 (−1.29; 4.49) ^1^	4.85 (−18.33; 34.63)	−14.70 (−29.40; −1.40)	−17.50 (−54.90; −2.80)
*p*	**0.044** ^2^	0.170 ^2^	0.272 ^2^	0.675	0.028	0.025
FIGO						
IA + IB	65.90 ± 12.05	75.86 ± 15.45	29.41 ± 5.95	54.98 (28.22; 101.57)	19.45 (15.65; 39.78)	71.85 (60.02; 99.15)
Inne	63.43 ± 13.28	77.95 ± 17.39	29.88 ± 7.01	85.84 (31.99;134.48)	20.60 (13.75; 137.10)	114.30 (73.48; 189.93)
MD (95% CI)	2.48 (−3.59; 8.54) ^1^	−2.09 (−10.09; 5.92) ^1^	−0.47 (−3.59; 2.65) ^1^	−30.86 (−53.57; 7.67)	−1.15 (−100.70; 10.30)	−42.45 (−104.90; 1.30)
*p*	0.420 ^2^	0.606 ^2^	0.764 ^2^	0.178	0.732	0.058
FIGO						
IA + IB	65.90 ± 12.05	75.86 ± 15.45	29.41 ± 5.95	54.98 (28.22; 101.57)	19.45 (15.65; 39.78)	71.85 (60.02; 99.15)
II	62.60 ± 15.77	83.80 ± 20.95	32.00 ± 8.24	64.97 (32.42; 97.63)	16.30 (13.10; 58.25)	77.30 (59.02; 104.28)
MD (95% CI)	3.30 (−5.09; 11.70) ^1^	−7.94 (−18.83; 2.95) ^1^	−2.59 (−6.80; 1.62) ^1^	−9.99 (−32.31; 27.35)	3.15 (−81.00; 45.20)	−5.45 (−31.70; 32.60)
*p*	0.436 ^2^	0.151 ^2^	0.224 ^2^	0.748	0.681	0.990
FIGO						
IA + IB	65.90 ± 12.05	75.86 ± 15.45	29.41 ± 5.95	54.98 (28.22; 101.57)	19.45 (15.65; 39.78)	71.85 (60.02; 99.15)
III	78.00 **	64.00 **	26.64 **	692.00 (692.00; 692.00)	-	-
MD (95% CI)	−12.10 (−36.28; 12.09) ^1^	11.86 (−19.18; 42.91) ^1^	2.77 (−9.18; 14.71) ^1^	−637.02 (−687.64; −382.35)	-	-
*p*	0.322 ^2^	0.448 ^2^	0.645 ^2^	0.092	-	-

Data were noted as mean ± standard deviation or median (first quartile; third quartile). MD—median difference ^1^ or median; CI—confidence interval. Group comparison was performed with the use of Mann–Whitney U and Student’s *t*-test for independent variables ^2^. * at the time of diagnosis. ** No standard deviation as there was only one patient in this subgroup.

**Table 7:** Diagnostic performance of FGF 21, CA 125, and HE4, accounting for tumor characteristics.

Variable	Cut-Off Point	AUC (95% CI)	Sensitivity	Specificity	Accuracy	PPV	NPV	*p*
Tumor histology (endometiroid vs. serous)								
FGF 21	23.10	0.597 (0.404;0.789)	0.21	1.00	0.29	1.00	0.13	**0.022**
Tumor histology (endometiroid vs. other)								
FGF 21	320.00	0.568 (0.390;0.746)	1.00	0.21	0.89	0.89	1.00	**0.023**
FIGO (IB vs. IA)								
HE4	68.05	0.683 (0.535;0.830)	0.58	0.76	0.65	0.79	0.53	**0.023**
FIGO (other vs. IA + IB)								
FGF 21	53.48	0.602 (0.522;0.683)	0.53	0.65	0.61	0.44	0.73	**0.004**

AUC—area under the curve, PPV– positive predictive value, NPV—negative predictive value. Only statistically significant results were demonstrated.

## Data Availability

The data presented in this study are available on request from the corresponding author. The data are not publicly available due to ethical restrictions.
